# Licensure of a Diphtheria and Tetanus Toxoids and Acellular Pertussis, Inactivated Poliovirus, *Haemophilus influenzae* Type b Conjugate, and Hepatitis B Vaccine, and Guidance for Use in Infants

**DOI:** 10.15585/mmwr.mm6905a5

**Published:** 2020-02-07

**Authors:** Sara E. Oliver, Kelly L. Moore

**Affiliations:** ^1^Division of Bacterial Diseases, National Center for Immunization and Respiratory Diseases, CDC; ^2^Department of Health Policy, Vanderbilt School of Medicine, Nashville, Tennessee.

On December 21, 2018 the Food and Drug Administration (FDA) licensed a hexavalent combined diphtheria and tetanus toxoids and acellular pertussis (DTaP) adsorbed, inactivated poliovirus (IPV), *Haemophilus influenzae* type b (Hib) conjugate (meningococcal protein conjugate) and hepatitis B (HepB) (recombinant) vaccine, DTaP-IPV-Hib-HepB (Vaxelis; MCM Vaccine Company),* for use as a 3-dose series in infants at ages 2, 4, and 6 months (*1*). On June 26, 2019, after reviewing data on safety and immunogenicity, the Advisory Committee on Immunization Practices (ACIP)^†^ voted to include DTaP-IPV-Hib-HepB in the federal Vaccines for Children (VFC) program.^§^ This report summarizes the indications for DTaP-IPV-Hib-HepB and provides guidance for its use.

## Introduction

Combination vaccines merge equivalent component vaccines into a single product to prevent more than one disease. The use of combination vaccines can reduce the number of injections patients receive and improve vaccine coverage rates (*2*,*3*). ACIP has previously stated that the use of a combination vaccine generally is preferred over separate injections of the equivalent component vaccines; considerations can include provider assessment, patient preference, and the potential for adverse events (*4*). Until 2018, there were two pentavalent combination vaccines licensed for use in the infant vaccine series: DTaP-HepB-IPV (Pediarix; GlaxoSmithKline) and DTaP-IPV/Hib (Pentacel; Sanofi Pasteur). In late 2018, a new hexavalent combination vaccine (DTaP-IPV-Hib-HepB) from the MCM Vaccine Company, a joint venture between Merck and Sanofi Pasteur, received FDA approval. Each dose of DTaP-IPV-Hib-HepB contains the same amount of diphtheria and tetanus toxoids and pertussis antigens (inactivated pertussis toxin [PT], filamentous hemagglutinin [FHA], pertactin, and fimbriae types 2 and 3) as does Pentacel. The poliovirus component of DTaP-IPV-Hib-HepB contains the same strains of inactivated poliovirus types 1, 2, and 3 as the poliovirus vaccine IPOL (Sanofi Pasteur), but in decreased amounts. The HIB component (Hib capsular polysaccharide polyribosyl-ribotol-phosphate [PRP] coupled to the outer membrane protein complex [OMP] of *Neisseria meningitidis*) is the same as that in PedvaxHIB (Merck), but in a decreased amount. The HepB component is the same as the pediatric formulation of Recombivax HB (Merck), but in an increased amount. The DTaP-IPV-Hib-HepB vaccine is a fully liquid formulation and requires no reconstitution.

## Methods

During December 2018–June 2019, the ACIP Combination Vaccines Work Group held monthly conference calls to review and discuss relevant scientific evidence regarding the inclusion of DTaP-IPV-Hib-HepB in the federal VFC program. The work group evaluated the relevant evidence related to the potential benefits and harms of DTaP-IPV-Hib-HepB. The new combination vaccine will not alter the established vaccination schedule, and no changes to current policy were discussed. At the June 2019 ACIP meeting, after discussion by ACIP members and a period for public comment, the ACIP members voted unanimously to include DTaP-IPV-Hib-HepB in the federal VFC program.

## Summary of Key Findings

Six Phase III studies evaluated the safety and immunogenicity of DTaP-IPV-Hib-HepB (*5*–*10*), including two noninferiority studies enrolling >4,200 children using the U.S. infant immunization schedule of 2, 4, and 6 months (*5*,*6*). The immunologic responses were assessed after the third dose of DTaP-IPV-Hib-HepB. Overall, the measured antibodies were noninferior to licensed comparator vaccines, with one exception: noninferiority was not met for the geometric mean concentration against one of five pertussis antigens (FHA) 1 month after completion of the 3-dose infant series. However, all pertussis antigens met noninferiority criteria for a second measured endpoint (the percentage that met a prespecified vaccine response). The DTaP-IPV-Hib-HepB vaccine had a safety profile consistent with that of the licensed component vaccines. A higher rate of fever was detected among DTaP-IPV-Hib-HepB recipients when compared with that among pentavalent vaccine (DTaP-IPV/Hib) recipients (47.1%–47.4% versus 33.2%–34.4%) (*5*,*6*). However, the rates of fever-related medical events, such as hospital visits or febrile seizures, were similar in the two groups.

Simultaneous administration of DTaP-IPV-Hib-HepB was tested with rotavirus and pneumococcal conjugate vaccines. Concomitant administration did not affect immunogenicity at measured endpoints for rotavirus (*5*). One of 13 pneumococcal serotypes, 6B, missed the prespecified noninferiority endpoint after the third dose (*6*). Pneumococcal serotype-specific correlates of protection are unknown, and it is unclear whether this would be clinically relevant. However, since the introduction of pneumococcal conjugate vaccines, pneumococcal serotype 6B is rarely detected in nasopharyngeal carriage or as a cause of invasive disease among U.S. children (*11*).

## Indications and Guidance for Use

DTaP-IPV-Hib-HepB is licensed for use in children aged 6 weeks through 4 years (before the fifth birthday) (Table) (*1*). DTaP-IPV-Hib-HepB is only indicated for use in infants at ages 2, 4, and 6 months.

**TABLE:** Recommended minimum ages for administration of DTaP-IPV-Hib-HepB vaccine and intervals between doses — United States, 2020*

Parameter	Age/Interval
Minimum age for any dose	6 weeks
Minimum interval between doses 1 and 2	4 weeks
Minimum age for dose 2	10 weeks
Minimum interval between doses 2 and 3	4 weeks
Minimum age for dose 3	24 weeks^†^
Maximum age for any dose	4 years, 364 days (do not administer on or after the fifth birthday)

**Abbreviations:** DTaP = diphtheria and tetanus toxoids and acellular pertussis; Hib = *Haemophilus influenzae* type b; HepB = hepatitis B; IPV = inactivated poliovirus.

* The DTaP-IPV-Hib-HepB vaccine (Vaxelis; MCM Vaccine Company) was licensed in December 2018 and will not be commercially available in the United States before 2021.

^†^ If the third dose of DTaP-IPV-Hib-HepB is given before age 24 weeks, an additional dose of hepatitis B vaccine should be given at age ≥24 weeks to complete the hepatitis B series.

For the prevention of diphtheria, tetanus and pertussis, children are recommended to receive a 3-dose primary series of DTaP, at ages 2, 4, and 6 months, and booster doses at ages 15–18 months and 4–6 years (*12*). DTaP-IPV-Hib-HepB can be used for the first 3 doses of the recommended DTaP series but should not be used for the fourth or fifth dose. However, if DTaP-IPV-Hib-HepB is inadvertently given for either booster dose, the dose does not need to be repeated with another DTaP-containing vaccine when the proper spacing of previous doses is maintained. Circumstances might warrant an accelerated schedule to provide early protection against pertussis, starting as soon as the infant is aged 6 weeks, with the second and third DTaP doses administered no earlier than 4 weeks after each preceding dose. The recommended minimum age for the third dose of the DTaP-IPV-Hib-HepB vaccine is 24 weeks, the minimum age for completion of the HepB vaccine series. Therefore, this combination vaccine is not recommended for use for the third dose of the primary series on an accelerated schedule at 4-week intervals for the prevention of pertussis.

For prevention of poliomyelitis, children are recommended to receive 4 doses of IPV, at ages 2, 4, 6–18 months, and 4–6 years (*13*). DTaP-IPV-Hib-HepB may be used for the first 3 doses of the IPV series but is not indicated for the fourth dose; however, if DTaP-IPV-Hib-HepB is inadvertently given for the booster dose, the dose does not need to be repeated with another IPV-containing vaccine, when the proper spacing of previous doses is maintained.

For prevention of invasive *H. influenzae* type b disease, children are recommended to receive a primary series (2 or 3 doses, depending on the vaccine used) of a Hib conjugate vaccine and a booster dose of vaccine at age 12–15 months (*14*). Although monovalent PRP-OMP Hib vaccines are licensed as a 2-dose primary series at ages 2 and 4 months, DTaP-IPV-Hib-HepB is licensed as a 3-dose primary series. Therefore, 3 doses of a Hib conjugate-containing vaccine are needed to complete the primary series if DTaP-IPV-Hib-HepB is used for any doses. DTaP-IPV-Hib-HepB should not be used for the booster dose (after completion of the 3-dose primary series). Any Hib conjugate vaccine licensed for a booster dose can be used. If DTaP-IPV-Hib-HepB is inadvertently given for the booster dose, the dose does not need to be repeated with another Hib-containing vaccine, when the proper spacing of previous doses is maintained.

For prevention of hepatitis B, children are recommended to receive 3 doses of a HepB vaccine at ages 0, 1–2, and 6–18 months, with variations depending on the maternal hepatitis B infection status, infant birthweight, and vaccine manufacturer (*15*). Universal HepB vaccination of all infants beginning at birth provides a critical safeguard and prevents infection among infants born to hepatitis B surface antigen (HBsAg)–positive mothers not identified prenatally. DTaP-IPV-Hib-HepB is not licensed for the birth dose but can be used for doses given at age ≥6 weeks to infants of HBsAg-negative mothers. In addition to this FDA-approved use, 3 doses of DTaP-IPV-Hib-HepB can be administered to an infant aged ≥6 weeks born to a woman who is HBsAg-positive or whose HBsAg status is unknown. For adequate immune response, the last dose of HepB vaccine should be given at age ≥24 weeks; therefore, the third dose of DTaP-IPV-Hib-HepB is not recommended to be given before age 24 weeks. If it is given earlier, an additional dose of HepB vaccine should be given at age ≥24 weeks, maintaining proper spacing with previous doses.

Data are limited on the safety and immunogenicity of interchanging vaccines from different manufacturers for the vaccination series in a child. Whenever feasible, the same manufacturer’s product should be used to complete the primary series; however, vaccination should not be deferred if the specific vaccine product previously administered is unavailable or unknown (*4*).

DTaP-IPV-Hib-HepB can be used for children aged <5 years requiring a catch-up schedule. However, vaccine doses should not be administered at intervals less than the minimum intervals provided in Table 3–1 of the General Best Practices Guidelines (*4*).

## Special Considerations

Before the routine use of Hib vaccines, incidence of *H. influenzae* meningitis among American Indian/Alaska Native (AI/AN) infants peaked at a younger age (4–6 months) than it did among other U.S. infant populations (6–7 months). Vaccination with a primary series of a Hib vaccine that contains PRP-OMP is preferred for AI/AN infants to provide early protection because these vaccines can provide a protective antibody response after the first dose (*13*). Data on antibody response after the first dose of DTaP-IPV-Hib-HepB in AI/AN infants are not currently available; therefore, DTaP-IPV-Hib-HepB does not have a preferential recommendation for AI/AN infants at this time. If data on antibody response after the first dose of DTaP-IPV-Hib-HepB become available, ACIP will re-evaluate the preferential language for the Hib component for AI/AN infants.

**Summary**

**What is already known about this topic?**

Combination vaccines merge equivalent component vaccines into a single product to prevent multiple diseases, which can reduce the number of injections administered and improve vaccination coverage.

**What is added by this report?**

A new hexavalent vaccine was approved by the Food and Drug Administration to prevent diphtheria, tetanus, pertussis, polio, *Haemophilus influenzae* type b, and hepatitis B (DTaP-IPV-Hib-HepB). At a recent Advisory Committee on Immunization Practices meeting, members voted unanimously to include this vaccine in the federal Vaccines for Children program.

**What are the implications for public health practice?**

The vaccine is licensed for use in children aged 6 weeks through 4 years and is indicated for the primary vaccination series in infants at ages 2, 4 and 6 months.

## Footnotes

* The manufacturer has stated that vaccine will not be commercially available in the United States before 2021.

† Recommendations for routine use of vaccines in children, adolescents, and adults are developed by the Advisory Committee on Immunization Practices (ACIP). ACIP is chartered as a federal advisory committee to provide expert external advice and guidance to CDC Director on use of vaccines and related agents for the control of vaccine-preventable diseases in the civilian population of the United States. Recommendations for routine use of vaccines in children and adolescents are harmonized to the greatest extent possible with recommendations made by the American Academy of Pediatrics (AAP), the American Academy of Family Physicians (AAFP), the American College of Obstetricians and Gynecologists (ACOG), and the American College of Nurse-Midwives (ACNM). Recommendations for routine use of vaccines in adults are harmonized with recommendations of AAFP, ACOG, the American College of Physicians (ACP), and ACNM. ACIP recommendations approved by the CDC Director become agency guidelines on the date published in the *Morbidity and Mortality Weekly Report* (*MMWR*). Additional information is available at https://www.cdc.gov/vaccines/acip.

§ https://www.cdc.gov/vaccines/programs/vfc/index.html.
